# Stroke unit demand in Norway – present and future estimates

**DOI:** 10.1186/s12913-021-07385-1

**Published:** 2022-03-15

**Authors:** Fredrik A. Dahl, Mathias Barra, Kashif W. Faiz, Hege Ihle-Hansen, Halvor Næss, Kim Rand, Ole Morten Rønning, Tone Breines Simonsen, Bente Thommessen, Angela S. Labberton

**Affiliations:** 1 The Health Services Research Unit (HØKH), Akershus University Hospital HF, Sykehusveien 25, 1478 Lørenskog, Norway; 2 Norwegian Computing Center, Oslo, Norway; 3 BCEPS, University of Bergen, Bergen, Norway; 4 Centre for Connected Care, Oslo University Hospital, Oslo, Norway; 5 Department of Geriatric Medicine, OUS, Oslo, Norway; 6 Department of Neurology, OUS, Oslo, Norway; 7 Department of Neurology, Haukeland University Hospital, Bergen, Norway; 8 Centre for Age-related Medicine, Stavanger University Hospital, Stavanger, Norway; 9 Institute of Clinical Medicine, University of Bergen, Bergen, Norway; 10 Institute of Clinical Medicine, University of Oslo, Oslo, Norway; 11 Department of Neurology, Akershus University Hospital, Lørenskog, Norway; 12 Norwegian Institute of Public Health, Health Services Research, Oslo, Norway

**Keywords:** Stroke unit, Stroke, Stroke mimics, Incidence, Length-of-stay, Health care management, Regression model

## Abstract

**Background:**

All stroke patients should receive timely admission to a stroke unit (SU). Consequently, most patients with suspected strokes – including stroke mimics (SM) are admitted. The aim of this study was to estimate the current total demand for SU bed capacity today and give estimates for future (2020–2040) demand.

**Methods:**

Time trend estimates for stroke incidence and time constant estimates for length of stay (LOS) were estimated from the Norwegian Patient Registry (2010–2015). Incidence and LOS models for SMs were based on data from Haukeland University Hospital (2008–2017) and Akershus University Hospital (2020), respectively. The incidence and LOS models were combined with scenarios from Statistic Norway’s population predictions to estimate SU demands for each health region. A telephone survey collected data on the number of currently available SU beds.

**Results:**

In 2020, 361 SU beds are available, while demand was estimated to 302. The models predict a reduction in stroke incidence, which offsets projected demographic shifts. Still, the estimated demand for 2040 rose to 316, due to an increase in SMs. A variation of this reference scenario, where stroke incidence was frozen at the 2020-level, gave a 2040-demand of 480 beds.

**Conclusions:**

While the stroke incidence is likely to continue to fall, this appears to be balanced by an increase in SMs. An important uncertainty is how long the trend of decreasing stroke incidence can be expected to continue. Since the most important uncertainty factors point toward a potential increase, which may be as large as 50%, we would recommend that the health authorities plan for a potential increase in the demand for SU bed capacity.

## Background

Timely admission to an SU reduces death and disability and increases the number of patients discharged home [1]. It is therefore imperative that health authorities adequately plan for future SU demand.

The delineation between transient ischemic attack (TIA) and ischemic stroke (IS) may have shifted over time [2], with increasingly sensitive imaging techniques [3]. In Norway, patients with TIA are admitted without delay to an SU for rapid work-up and start-up of secondary prevention [4]. Hence, the hospitalisation of TIA patients, as opposed to out-patient treatment, increase the need of SU beds. Further, patients admitted to the SU for suspected stroke, but without confirmed stroke or TIA at discharge—so-called ‘stroke mimics’ (SM)—might represent up to 50% or more of stroke-like presentations and must also be accounted for in SU capacity planning [5–7].

Although declining stroke incidence rates have been observed in high-income countries over the last 30 years, the trend in absolute numbers is offset by an aging population [8–10], and more liberal admission guidelines [3, 11]. Recent estimates of incidence rates in Norway have found a decline in age- and sex-adjusted incidence for IS and non-significant trends for intracerebral haemorrhage (ICH) and TIA during the years 2010–2015 [12, 13]. In addition, there appears to be a general reduction in the average length of stay (LOS) for stroke patients [11]. On the other hand, similar reductions in admission rates for SM are not found [7, 14, 15].

Previously, our research group has published time trend estimates for the incidence of IS, ICH, and TIA, estimated LOS for stroke patients on SUs, and incidence of SU admissions for SMs. The aim of the present study is to integrate our previous findings with demographic projections in order to estimate the demand for SU beds in Norway for the years 2020–2040. We thereby account for trends in incidence rates for stroke and TIA, demographic projections, accounting for the effects of LOS and utilisation of SU beds by patients with SMs [7, 12, 13].

## Methods

### Study setting

**Fig. 1:**
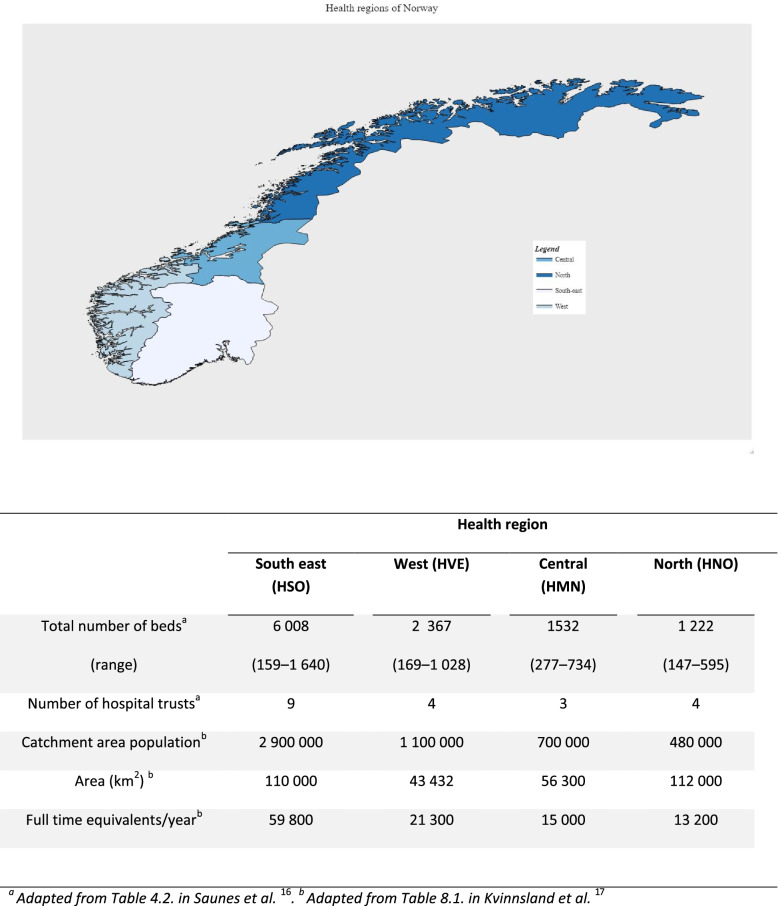
Map displaying the Norwegian Health Regions, with key characteristics [17]

Norway provides universal healthcare to all residents, and patients with suspected stroke are exclusively admitted to public hospitals. Hospital care is the responsibility of four Regional Health Authorities each responsible for one (geographical) health region; see Fig. 1. The number of hospital trusts and beds in each region reflects population size, with the greatest number in the most densely populated South-East region. The number of beds at the hospital trusts are generally in the range 160–1600. The health regions differ in geography and population density, see Fig. 1. For example, in the North region, the distance between hospitals can exceed 500 km. For a comprehensive introduction to the Norwegian health system, see Saunes et al. [16]

### Data sources

We used the following data sources to answer the research questions:

#### Norwegian Patient Registry (NPR)

The NPR contains information on all treatment-related activity in Norwegian public hospitals, covering all admitted strokes. Suspected acute stroke or TIA are exclusively admitted to public hospitals in Norway. Data from the period 2010–2015 was used to estimate stroke and TIA incidences and LOS.

#### NORSTROKE

This registry has prospectively registered all admissions to Haukeland University Hospital’s SU since 2008 until the present [18]. For this study, analyses of ~ 6000 SM admissions between 2008 and 2017 have been used to estimate temporal trends in SM-incidence. NORSTROKE did not contain LOS for SM admissions [7].

#### Statistics Norway (SN)

We extracted demographic projections (February 2021), or scenarios, for population size and composition, by sex, yearly age, and municipality for 2020–2040 with low, medium, and high aging [19].

#### The Electronic Patient Journal (EPJ) at Akershus University hospital

To estimate SM LOS we extracted LOS for all SU-admissions 2020 for patients admitted to the Akershus University Hospital SU (*n = 1380*). The resulting SM LOS estimate was then validated against a small (*n = 50*) random sample from the SU at Haukeland University Hospital (NORSTROKE).

#### Current SU bed capacity data

A telephone survey to all 49 Norwegian hospitals treating patients with acute stroke was conducted during May 2020. We recorded data about the number of beds allocated to acute stroke during normal operating levels, and in which department/unit the beds were located, in addition to information about additional bed-usage in the case of overcrowding. With this survey we were primarily interested in the number of beds allocated to treating patients with acute stroke, without any formal assessment of whether these beds satisfied criteria for an SU. One hospital operates purely as a tertiary-referral hospital for performing thrombectomy and neurosurgical procedures before transfer back to the treating hospital. The beds allocated stroke patients in this hospital are therefore not counted in the total number of beds available in Norway. Three hospitals were unable to give a specific number for stroke patients, only a total number for the ward which was shared with other patient groups. For these three hospitals we estimated the number of beds based on number of strokes they reported to the Norwegian Stroke Registry 2014–2018 and compared these to hospitals with a similar number of annual patients and for which a number for beds were specified.

### Model parameters

#### Diagnoses

Our analyses employ the diagnosis categories ICH (intracerebral haemorrhage¸ I61), IS (ischemic stroke; I63), and TIA (transient ischemic attack; G45, excluding G45.3 and G45.4). In addition, any patient admitted to an SU where the discharge diagnosis was neither IS, ICH, nor TIA was classified as a SM. Although the last category covers various and diverse diagnoses, we treat it as a single category in our models and define the set of diagnoses *D* = {*ICH*, *IS*, *TIA*, *SM*}.

#### Geographical regions

Norway is divided into four health regions (*R*): South-east (HSO), West (HVE), Middle (HMN) and North (HNO): *R* = {*HSO*, *HVE*, *HMN*, *HNO*}.

#### Age and sex

We used integer age ranging from 18 to the maximal recorded age in our data sets; *A* = {18, 19, …, 105}. Sex was classified as male or female: *S* = {*m*, *f*}.

#### Time period

The models give forecasts for the years *Y* = {2020, …, 2040}.

#### Demand

The dependent variable of the models are the predicted Demands, defined as the average number of SU-beds in use at midnight in a given year and geographical area.

### Component models

#### Stroke incidence

The stroke incidence models were modified versions of the models from Rand et al. [13] Poisson regression was used [20], with dependent variable ‘the expected number of hospitalisations per person-year’. The NPR data included the diagnosis ‘I64’ (undetermined type), which was analysed separately by Rand et al. However, as that analysis showed, this diagnosis had low and sharply declining incidence. In the present analysis these cases were randomly recoded as either IS or ICH, according to the relative incidence (IS:ICH = 85:15) of these diagnoses in the NPR dataset.

Separate regression models were developed for the diagnoses, each with the predictors age, sex, region, and year. We did consider estimating separate models also for each region, but the annual variability of the incidence in the different regions was too high to support such models. The chosen models included sex, age, age-squared, year, and health region. The functional form for the expected number of admissions in year *y* for a person of age *a* and sex *s* residing in region *r* is given by: $\exp \left({\beta}_0+{\beta}_ss+{\beta}_aa+{\beta}_{a^2}{a}^2+{\beta}_yy+{\beta}_r\right)$, where the exponential function ensures positive estimates. When the regression is used to estimate the number of admissions, the expression is multiplied by the forecasted number of inhabitants of age *a* and sex *s* in region *r* in year *y*.

#### Stroke LOS

We did not consider the time-of-day registrations for admissions and discharges accurate enough to be used in the computations and therefore used a time resolution of ‘days’, defining LOS = discharge date – admission date, which is equivalent to counting the number of midnights the patient was admitted. This rounds off the hours in a way that overestimates the time spent for patients that were admitted in an evening and discharged in a morning, and vice versa. Note that a LOS of zero is thus possible.

Poisson regression was used also for the LOS models. Sex was not statistically significant for any of the diagnoses, and the final model included age, age-squared, year, and region. Although the model included a year effect, this was only used for estimating the LOS for the last year of the NPR data set (2015), and these estimates were used in the main prediction runs of the model. The reason for this methodological choice was that the downward trend in LOS was primarily seen as a reactive adjustment made by the hospitals, rather than a change in the medical needs of the patients.

#### SM incidence

SM incidence was based on Barra et al. [7], We follow Barra et al. in being careful in concluding on whether the temporal trend has plateaued or if it will continue to increase going forward.

#### SM LOS

We used Akershus University Hospital’s EPJ data to estimate SM LOS with the same specification used for stroke, but without region as a covariate. We therefore evaluated Poisson regression models with age, age-squared, and sex. Of these predictors, only age had a statistically significant effect and was included in the model.

#### Demographics models

The population projections use SN’s scenarios (*X*) to estimate demands given low, medium, or high ageing. The SN scenarios’ four-letter codes (e.g. HLMH) represent low (L), medium (M), or high (H) level on four main drivers of demographic development: fertility, life expectancy, domestic migration (inter-regional relocation), and immigration. The three scenarios chosen for our analysis are dubbed by the SN as the ‘main’ (M**M**MM), ‘low ageing’ (H**L**MH) and ‘high ageing’ (L**H**ML) alternatives [19]. We abbreviate these here by their second (boldface) letter, as this reflects the scenario’s assumptions about longevity: *X* = {*M*, *L*, *H*}. For example, L**H**ML stipulates ‘high longevity’, hence an increase in the number of high-risk-for-stroke individuals, which is what we are exploring.

### Combined model

The incidence model has the form *I*(*a*, *s*, *r*, *y*, *d*), where *a* ∈ *A* is age, *s* ∈ *S* is sex, *r* ∈ *R* denotes region, and *y* ∈ *Y* is year. *d* ∈ *D* is the diagnosis, and while we conceptually regard this as one specification, we performed a separate regression for each diagnosis. Similarly, the LOS model has the form *L*(*a*, *s*, *r*, *y*, *d*). The demographics model has the form *n*(*a*, *s*, *r*, *y*, *X*), where the output is the predicted number of inhabitants of age *a* and sex *s* in region *r* in year *y.* The variable *X* denotes SN-scenario ({*M*, *L*, *H*}).

The expected number of midnights that a person of age *a* and sex *s* stays in an SU in region *r* in year *y* due to diagnosis *d* is thus estimated by the product *I*(*a*, *s*, *r*, *y*, *d*) × *L*(*a*, *s*, *r*, *y*, *d*). The predicted average number of SU overnight-stays in a region *r* in year *y* due to diagnosis *d* is given by aggregation over age and sex (relative to demographic scenario *X*)*,* divided by 365, to give the predicted average number of beds in use on any given day: *the demand*

1 $$\mathrm{Demand}\left(r,y,d,X\right)=\frac{\sum_{a\in A,s\in S}I\left(a,s,r,y,d\right)\times L\left(a,s,r,y,d\right)\times n\left(a,s,r,y,X\right)}{365}$$

For example, Demand(*HNO*, 2030, *TIA*, *M*) denotes the predicted average number SU beds occupied in health region North (*r* = *HNO*), by a TIA patient (*d* = *TIA*), averaged over midnights in 2030 (*y* = 2030), under the main demography scenario (*X* = *M*).

Our reference scenario assumed: demographics alternative *M*, a continued decline in stroke incidence as forecast by our models, and no time trend in LOS, i.e. 2015-levels for LOS. We also explored deviations from this scenario in terms of alternative demographic forecasts ‘High aging’ and ‘Low Aging’, and for a scenario in which stroke incidence does *not* continue to decline.

All analyses were performed using the statistical software R, version (3.6.1) [21], and were carried out, to the best of our knowledge, in accordance with best practices. Ethical approval was either obtained or waived by the relevant authorities (local ethical committees or privacy officers,) in accordance with Norwegian legislation. Where applicable, informed consent was obtained from all subjects/legal guardian(s). Only publicly available data (SN), registry data (NPR), or administrative data from the authors’ home institutions (HUS/Ahus) were used for this study, and neither surveys nor patient journal data was used; see also the Ethics section preceding the reference list.

## Results

General summary statistics of the input data sets are provided in Table 1. Note that all estimates for stroke (including TIA) were based on a data set covering all of Norway between 2010 and 2015. The estimates regarding stroke mimics were based on single-hospital data as outlined above.

**Table 1:** Descriptive summary statistics for the various data sets utilised: *Ahus* data set from Akershus University Hospital used to estimate SM LOS (obtained for the present study), *HUS* data set from NORSTROKE/Haukeland University Hospital used to estimate SM incidence [7], *NPR* data set from Norwegian Patient Registry used to estimate TIA, ICH and IS incidences and LOS [7, 13], *SM* Stroke mimics, *ICH* Intracerebral haemmorhage, *IS* Ischemic stroke

	Descriptive summary				
*Data set*	*Ahus*	*HUS*	*NPR*		
Time Period	2020	2008–2017	2010–2015		
Diagnosis	SM	SM	TIA	ICH	IS
Number of admissions	1380	5732	28,138	10,011	57,626
Male %	49	46	50	53	54
Mean age (years)	61	68	72	73	74
Mean LOS (nights)	2.4	NA	2.5	8.3	6.9

The regression coefficients for the incidence and the LOS models are given in Table 2.

**Table 2:** Incidence and Length of stay model coefficients>

*Variable*	*TIA*	*IS*	*ICH*	*SM*
**Incidence**				
Intercept	−15.385	−14.005	−14.070	−9.403
Sex	0.234***	0.453***	0.405***	0.047 ***
Age	0.200***	0.164***	0.119***	0.047***
Age^2^/100	−0.091***	−0.056***	−0.028***	−0.009***
Year^a,b^	−0.022***	−0.045***	−0.016***	NA
Health Region^c^				
Central	0.138***	−0.074***	−0.107***	NA
North	0.092***	−0.088***	0.048	NA
West	−0.258***	−0.229***	−0.087**	NA
**Length of stay**				
Intercept	1.226	2.105	1.690	0.419
Age	−0.012***	−0.007***	0.029***	0.007***
Age^2^/100	0.011***	0.006***	−0.029***	NA
Year^a^	−0.015***	−0.043***	−0.053***	NA
Health Region^c^				
Central	−0.139***	0.131***	0.072***	NA
North	0.004	0.317***	0.165***	NA
West	0.202***	0.236***	0.040***	NA

^a^Year 2010 set to 0 in all models

^b^For the SM model, no temporal trend was be estimated

^c^South-east is reference region

* < 0.05, ** < 0.01, *** < 0.001

As outlined in Methods, extrapolation of temporal trends for LOS for stroke patients was not used in the combined analysis, and the year predictor was frozen at the last year of the data set (2015). For 2020 this model predicts a national average LOS for the different diagnoses, as TIA: 2.4, IS: 6.2, ICH: 7.3, and SM: 2.5. Since a temporal trend is not used, there are only slight changes to these estimates for the different population scenarios, due to shifts in age and sex distribution.

### Current status (2020)

All the 49 contacted hospitals reported on SU capacity. Figure 1 shows a map of the health regions and selected characteristics. The numbers of beds per region were: South east = 182, West = 68, Central = 51 and North = 60; a total of 361 beds.

### Predictions 2020–2040

Table 3 gives the estimated demands for 5-year intervals from 2020 to 2040, under the reference scenario assumptions of medium demographic development, continued time trend of stroke incidence, and stroke patient LOS frozen at 2015-level. For 2020, the scenarios have not yet diverged, so there is only one prediction (scenario *M*). We observe that for South-east, the average bed utilisation is estimated to 92% of available capacity, while for West, Middle, and North the corresponding figures are 85, 84 and 57%. The national estimate is 84%.

**Table 3:** Predictions of demand by region and diagnosis in 5 years intervals. In each cell the numbers give the estimated demand in the SN scenarios L = low aging, M = reference scenario, H = high ageing, and M(F) = reference scenario with frozen stroke incidence at 2020-level. Reference scenario estimates are in bold. (For year = 2020 there is no scenario) For each health region, the currently available beds are provided in the column ‘Available’.

Year	2020		2025	2030	2035	2040
**Region**	**Available**	**M**	**L/M/H/M(F)**	**L/M/H/M(F)**	**L/M/H/M(F)**	**L/M/H/M(F)**
Norway	361	**302**	300/**303**/306/340	302/**308**/315/386	303/**314**/324/435	301/**316**/331/480
South east	182	**167**	158/**166**/168/188	168/**171**/175/213	169/**175**/181/241	169/**177**/185/267
West	68	**58**	58/**59**/59/66	59/**60**/61/75	60/**62**/64/85	60/**62**/65/94
Middle	51	**43**	43/**43**/44/49	43/**43**/45/55	43/**44**/46/62	42/**44**/46/67
North	60	**34**	33/**33**/34/38	33/**33**/34/43	32/**33**/34/47	31/**33**/34/51
**Diagnosis**						
IS	NA	**135**	121/**122**/124/153	110/**113**/115/176	99/**103**/107/200	86/**92**/97/224
SM	NA	**103**	113/**114**/115/114	125/**127**/130/127	138/**142**/146/142	148/**155**/162/155
ICH	NA	**34**	35/**35**/36/38	36/**37**/37/43	36/**38**/39/48	36/**38**/39/53
TIA	NA	**31**	31/**31**/32/35	31/**32**/32/39	31/**32**/33/44	30/**32**/33/49

**Fig. 2:**
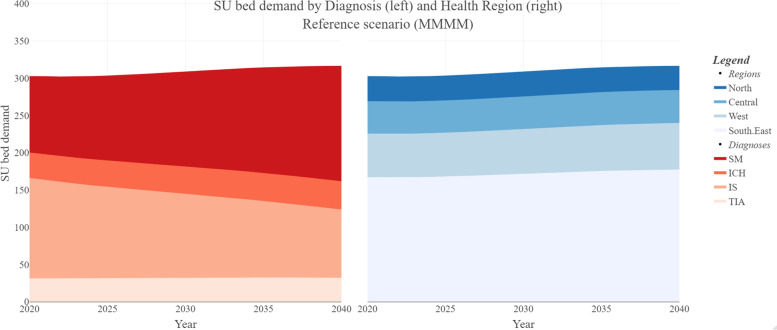
Average number of stroke unit beds in demand for reference scenario (M), stratified by diagnosis (left) and health region (right)

Figure 2 shows the predicted demands by diagnosis and by region for the 2020–2040 period for the reference scenario (demographics alternative *M*, a continued decline in stroke incidence as forecast by our models, and no time trend in LOS.)

**Fig. 3:**
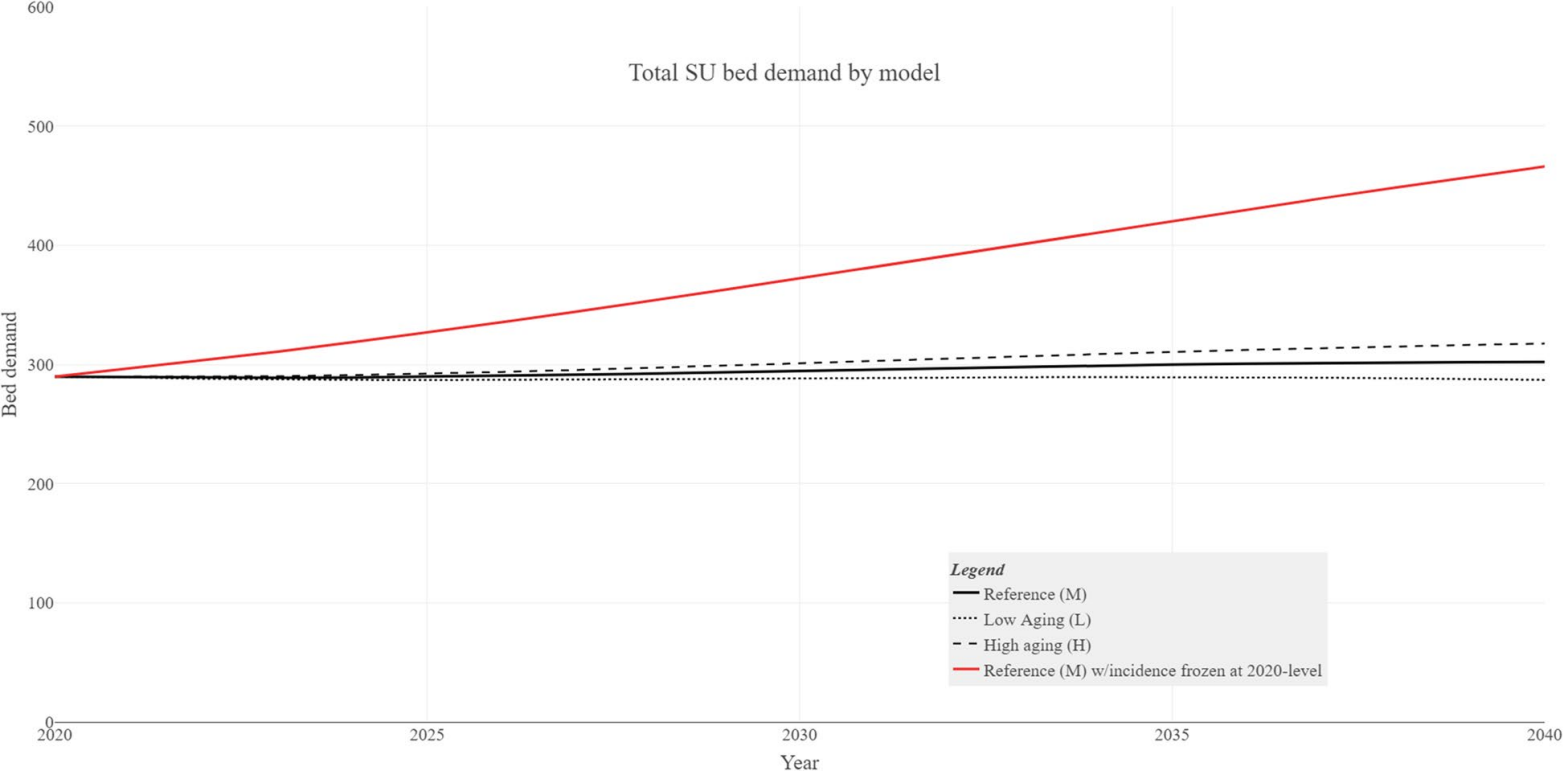
National estimated demand for stroke-unit beds by demographic and incidence scenario variations

Figure 3 shows the graphs for the national predicted demands for the different demographic scenarios.

### Sensitivity analysis

The implications of a continued decrease in LOS can be inferred from the coefficients given in Table 2. The age specific LOS would have an annual reduction of 1 − exp(−0.015) = 1.4% for TIA, 4.0% for IS, and 5.2% for ICH, which over a period of 20 years implies reductions of 24, 56 and 65%, respectively.

The predictors that are related to time have the largest impact on the estimated demands. If the set of predictors is reduced to age and year only – i.e., excluding sex, age [2], and health region from the incidence models, and sex from the LOS models – the estimated 2040 national demand changes by only four beds in the reference scenario. If age is also omitted, the estimate changes dramatically to 200, while the impact of the time trend variable (year) is shown in Fig. 3.

Poisson regression was used for each of the component models, which is possible because the number of admissions and the number of LOS days are represented as non-negative integers. In some applications, negative binomial regression is preferred over Poisson regression because the latter handles so-called over- or under-dispersion less naturally. This is mainly related to the standard errors of the parameter estimates and the width of confidence intervals, which are less important for prediction applications. We also tested negative binomial models, and in several cases, they were numerically unstable. We have therefore chosen the more robust Poisson regression throughout.

## Discussion

The calculated demand for 2020 corresponds well with the current capacity, with higher SU bed utilisation in densely populated areas, which is expected due to lower random fluctuations in demand. The baseline scenario shows that the predicted reduction in stroke incidence more than compensates for the population growth and ageing. The net reduction in strokes is replaced by a predicted increase in SMs, resulting in a slightly higher demand for SU beds in 2040 compared to 2020 (316 vs. 302).

This study indicates that the future demand for SU beds is uncertain and extremely sensitive to whether the downward trend in stroke incidence continues. There is also uncertainty in future trends of SM incidence. Our predictions should be considered conservative, since we assume that stroke continues to decline, at the same time as the incidence of mimics does not increase.

The estimated occupancies for 2020 fit the reported capacity reasonably well. Despite random fluctuations, an average demand of 84% of the capacity would imply that most admissions can be handled by a local SU. A level of 92% for the region South-east may be problematic, but this region has larger units, which are less vulnerable to random fluctuation: a low variance in admissions makes it possible with higher average occupancy. In the Oslo area (within region South-east) there are several SUs, which – in theory – adds flexibility in SU utilisation. For the North region, the estimated 2020 average occupancy was substantially lower (57%), which is reasonable by the contrapositive argument: this region has low population density, covers a large geographical area, and smaller SU units, which necessitates a relatively lower average occupancy to accommodate variance in the number of admissions. We note that The Norwegian Stroke Organisation performed their own survey of SU capacity in 2020 that reported 362 beds [22], which differs slightly from our counts.

The analysis of the reference scenario shows a relatively stable demand until 2040, nationally and within regions, due to four interacting trend-assumptions:

1. falling incidence of stroke, in particular ischemic stroke;
2. stable incidence of SMs;
3. stable length-of-stay;
4. a growing and ageing population.

The impact of deviations from assumption (1) is illustrated by Fig. 3, and the analysis shows a 50% increase in the demand compared to the reference scenario if the stroke incidence is frozen at the 2020 level [13]. discusses this issue, and we agree that there is a fundamental uncertainty regarding the duration of the favorable cardiovascular trend in the population. From a modelling point of view, however, it seems likely that a possible change in the trend will happen gradually, and not take full effect until late in the period.

Regarding assumption (2), the physiological mechanisms of SM are largely independent of cardiovascular risk factors, and there is no evidence of a falling incidence trend. On the contrary, Barra et al. [7] show an increasing trend in incidence of hospitalisations for SMs between 2008 and 2017, but conclude that the trend appears to flatten out near the end of that period; an assumption we have incorporated in our models. If SM incidence continues to rise, however, a substantial increase in demand would result. The findings from this study are somewhat higher than reported in another Norwegian study, which reported a share of SMs of 38% in 2012 [5, 7], and is substantially higher than found in many studies from other countries [14, 15, 23, 24]. None of these report on time-series, however, and we are not aware of any recent studies that contradict the strong increasing trend of SU admittance for SMs between 2008 and 2017. We remark here that the incidence of admittance to an SU with SMs in our forecasts is based on a study from *only one* (large) Norwegian hospital and may be higher there than for other populations. There is, however, no evidence that Haukeland University Hospital’s admittance policies differ in a systematic way from other Norwegian hospitals. (Indeed, at Akershus University Hospital, we were able to check during the review process to confirm that in 2020 the share of SMs surpassed 50%).

The large share of hospitalisations due to SMs – even if overestimated here – highlights the importance of pre-hospital or early screening and rapid work up. We hope advances are made in the diagnostic pathway for suspected strokes, resulting in better specificity, so that fewer SMs are admitted to the SU in the future. Given such advances, our estimates will overestimate the demand for SU beds.

Assumption (3) is in fact contrary to empirical observations: the NPR stroke dataset shows a downward trend in SU LOS. However, if we extend this trend through to 2040, the average LOS for ICH patients would fall to 2.5 days, which is very unreasonable and medically indefensible [1]. There are several reasons why we choose to keep the LOS stable in the analysis: Importantly, the current average LOS is consistent with the recommended shortest SU LOS for stroke patients from Norwegian national guidelines for standards of care, and further reductions are not advisable [25]. Second, the period 2010–2015 coincided with the implementation of the Norwegian *Coordination reform*, which included measures designed to reduce (excess) hospital LOS. Specifically, the reform introduced a substantial remuneration from the municipalities to the hospitals if they were unable to receive patients declared ready for discharge. Third, we consider the changes in LOS largely as an endogenous variable, which captures the hospitals’ responses to changes in the demand. We find it most informative to make predictions under the assumption that the level of care is kept constant. Still, there are uncertainties in this area also, and it may be possible to reduce the LOS slightly without compromising the level of care. In addition to a trend of fewer strokes, there is also a trend toward milder strokes [11], which may lead to shorter SU LOS in the future. Also, for SM patients, the SU stays are mostly about diagnostics, and technical progress in medical imaging may reduce their LOS.

Assumption (4) is least problematic, although the demographic trends explain nearly 50% of the increase in demand when stroke incidence is frozen. The SN scenarios of high and low ageing project changes in 2040 occupancy of approximately 5% up or down from the reference scenario, which is substantial, but still moderate compared to the other uncertainties discussed above. The SN scenarios represent internal migration, but the results in Table 3 show no important between-region effects.

There is a specific demographic uncertainty for the catchment area of Akershus University Hospital, however, which has a substantial number of immigrants that are suspected to have an elevated stroke risk [26]. At present, this group is relatively young, but during the forecasted period these individuals will shift into high-risk-for-stroke age-groups. We also note that although evidence from Norway suggest elevated stroke-risk in some immigrant groups, the opposite might be true. In neighboring Denmark, a contemporary study reported *lower* stroke incidence for immigrants after adjusting for sociodemographic covariates [27]. We have not estimated the magnitude of such effects.

Sensitivity analyses of the predictors included in the component models show that only age and year have an impact on the predictions that is large enough to be important for policy makers. It may be surprising that a predictor like sex, which has a strong effect on incidence, has little impact on the overall demand. The explanation is that an incidence model without sex as a predictor will make unbiased average estimates over the whole baseline population, and if the sex distribution in the population remains relatively stable, the aggregate estimates remain sound. The two main drivers in the predictions are a) the population aging combined with a strong age gradient in incidence and b) the time trend in incidence. It thus stands to reason that age and year affect estimates the most.

The main strength of the present study is the integration of separate state-of-the-art models for incidence and LOS for SU patients in Norway, combined with approved demographic scenarios. The models for stroke and TIA diagnoses are based on nationwide data. For other model inputs we include data from several hospitals. Within the model suite, the SM models are the least general since these are estimated from smaller datasets that do not cover the whole country. Still, and as always, the main limitations of the study are given by fundamental uncertainties in the extrapolation of the historic trends.

## Conclusions

A slightly higher average SU bed demand is predicted in 2040 compared to today under the reference scenario. A reduction in numbers of strokes, particularly ischemic stroke, is predicted despite expected population growth and ageing. An increase in stroke mimic admissions replace the net reduction in strokes and TIAs. The main uncertainties to our predictions relate to future incidence trends for both stroke and stroke mimics.

Since the most important uncertainty factors point toward a potential increase, which may be as large as 50%, we would recommend that the health authorities plan for a potential increase in the demand for SU bed capacity. At the very least, we would strongly advise against a reduction in this important capacity before such uncertainties are substantially reduced.

## Data Availability

The data that support the findings of this study are available on reasonable request from the corresponding author. The data are not publicly available due to privacy or ethical restrictions.
